# When symptoms don’t fit: a case series of conversion disorder in the pediatric otolaryngology practice

**DOI:** 10.1186/s40463-018-0286-7

**Published:** 2018-05-29

**Authors:** Lisa Caulley, Scott Kohlert, Hazen Gandy, Janet Olds, Matthew Bromwich

**Affiliations:** 1 Faculty of Medicine, University of Ottawa, Ottawa, ON Canada; 2 Department of Otolaryngology-Head and Neck Surgery, The Ottawa Hospital, Ottawa, ON Canada; 3 Division of Otolaryngology – Head and Neck Surgery, Children’s Hospital of Eastern Ontario, 400 Smyth Road, Ottawa, ON K1H 8L1 Canada; 4 Department of Psychiatry, Children’s Hospital of Eastern Ontario, University of Ottawa, Ottawa, ON Canada; 5 Department of Psychology, Children’s Hospital of Eastern Ontario, University of Ottawa, Ottawa, ON Canada

**Keywords:** Pediatrics, Conversion disorders, Otolaryngology, Misdiagnosis

## Abstract

**Background:**

Conversion disorder refers to functional bodily impairments that can be precipitated by high stress situations including trauma and surgery. Symptoms of conversion disorder may mimic or complicate otolaryngology diseases in the pediatric population.

**Case presentation:**

In this report, the authors describe 3 cases of conversion disorder that presented to a pediatric otolaryngology-head and neck surgery practice. This report highlights a unique population of patients who have not previously been investigated. The clinical presentation and management of these cases are discussed in detail. Non-organic otolaryngology symptoms of conversion disorder in the pediatric population are reviewed. In addition, we discuss the challenges faced by clinicians in appropriately identifying and treating these patients and present an approach to management of their care.

**Conclusion:**

In this report, the authors highlight the importance of considering psychogenic illnesses in patients with atypical clinical presentations of otolaryngology disorders.

## Background

The prevalence of mental illness is estimated to be 10–20% amongst children and adolescents worldwide, making it the leading cause of disability in young people [1]. Furthermore, treatments (both behavioral and pharmacological) of mental illness and the demand for them for children and adolescents has increased significantly in the past decade [2]. Untreated psychiatric disorders can impair a child’s development and limit educational achievement [1].

Conversion disorders refer to body dysfunction characterized by neurological symptoms, either sensory or motor, that cannot be explained by a medical condition. Given their somatosensory nature, they typically require a medical assessment and the diagnosis of conversion disorder can only be established after organic diseases have been excluded or if they fail to account for the severity of a patient’s impairment. In pediatric patients, the presentations of conversion disorder tend to be complex, and multiple conversion symptoms are the norm [3–5]. As it has been found to be associated with bodily stress, it is imperative that surgeons are aware of this disorder in the post-operative setting [5–9]. Developing an approach to this issue requires an appreciation for the multifactorial nature of its etiology.

It is prudent that clinicians be informed about the prevalence of mental illness in their patient population and its implications. Misdiagnosis or delayed diagnosis can have a significant impact on patients and creates a burden not only on the healthcare system, but also on the patient and their family members. In this article, the authors discuss 3 pediatric cases referred for otolaryngologic complaints that were complicated by conversion disorder. We discuss the implications of conversion disorder for the diagnosis and treatment for the otolaryngologist - head and neck surgeon and the need for an awareness of the impact of conversion disorders on presentation, treatment and recovery.

## Case presentations

Patient 1 was a previously healthy 11-year-old girl who presented to hospital with a 2-week history of “dizziness”. Her symptoms were described as disequilibrium precipitated by standing and sitting and relieved by lying flat. Her symptoms were unaffected by eye opening. Her symptoms were debilitating and she had difficulty ambulating. Her symptoms were unresponsive to antiemetics and she presented to the Children’s Hospital of Eastern Ontario emergency room where she was diagnosed with vestibular neuronitis. When her symptoms persisted, she was admitted to hospital and assessed by the Otolaryngology-Head and Neck Surgery service. In addition to a history and focused head and neck examination, an oto-neurological examination was performed including evaluation of cranial nerves, voluntary saccades, spontaneous and gaze-evoked nystagmus, rapid head thrust and dix-hall pike maneuver. She did not demonstrate any clinical findings suggestive of vestibular neuronitis, migraine variant nor benign positional paroxysmal variant. Routine laboratory investigations were within normal limits. Magnetic resonance imaging of the brain was non-contributory. She was admitted to hospital for 4 days. She received instructions for daily strengthening exercises from the physiotherapy service. These interventions validated her experiences and offered a mechanism for symptom resolution that was psychologically and emotionally acceptable to the patient and her family, which resulted in a complete resolution of her symptoms. A previous diagnosis of social anxiety some 5 years ago may have been a relevant risk factor in the development of her symptoms.

Patient 2 was an 11-year-old girl who presented to hospital with a history of head trauma while somersaulting 3 weeks prior to her presentation. She described progressive headaches, disequilibrium, choreiform movements and ataxia following the mild traumatic head injury. Her symptoms were debilitating and she was unable to sit upright or ambulate. She had no significant past medical history. She was admitted to the medical service for 23 days, during which she was assessed by multiple subspecialties including the Otolaryngology – Head and Neck Surgery service. After a complete oto-neurological examination, she was found to have no evidence of a vestibular pathology to account for her symptoms, and a head computed tomography (CT) and magnetic resonance imaging (MRI) were normal. Routine laboratory investigations were within normal limits. Further assessment from Mental Health services identified the following contributing factors: post concussive symptomatology including high anxiety, high family expectations in the presence of limited communication and sibling rivalry, and the presence of a pre-existing significant traumatic event. A diagnosis of conversion disorder was made, and conceptualized as an unconscious avoidant coping mechanism. While an inpatient, she was followed by a multidisciplinary team including Psychiatry, Psychology and Physiotherapy. The focus of mental health interventions was on communication and expression of emotion, while Physiotherapy provided exercises to improve her symptoms and validation of her psychological distress. She improved significantly over the course of hospitalization and was discharged to outpatient follow-up through Mental Health Services for continued intervention and support.

Patient 3 was a 13-year-old boy who underwent an adenoidectomy. He had a past medical history of significant nasal obstruction due to adenoid hypertrophy. His post-operative course was complicated by recurrent adenoid bleeds. One month post-operatively, the patient began to complain of daily headaches. Over the two weeks following, he reported daily nausea and disequilibrium. He returned to the emergency department when he developed complete paralysis of the lower limbs, essentially rendering him paraplegic and disabled. A complete head and neck examination including oto-neurologic examination was within normal limits. Routine laboratory investigations were within normal limits. An MRI and MR venogram of the brain failed to reveal evidence of any intracranial pathology. He remained in hospital for 17 days. His gait progressively improved with physiotherapy until he returned to baseline. The Mental Health service identified pre-existing significant traumatic events and psychosocial stressors for the entire family associated with multiple moves and living in a refugee camp for a year and a half prior to immigrating to Canada. In addition, the patient was found to have some evidence of anxiety and perfectionism that were exacerbated by his inability to participate in school, secondary to the surgery.

## Discussion

To our knowledge, this is the first reported case series of pediatric psychiatric disorders that presented for consideration of otolaryngology-related pathology. Mental health disorders are misdiagnosed as organic diseases more frequently than clinicians expect due to several disease, patient and clinician factors. Furthermore, the ability to accurately diagnose patients with a psychiatric illness may often fall outside of the scope of practice for the average Otolaryngologist – Head and Neck Surgeon. However, as highlighted in the following section, it is crucial that clinicians keep psychiatric illnesses on the differential diagnosis, especially for patients who present with atypical or contradictory physical signs and symptoms. In addition, an approach to the management of these patients is provided as a resource for clinicians.

Functional disorders have been linked in the adult literature with a wide breadth of head and neck complaints, such as hearing loss, anosmia, stridor, dysphonia, and vision loss [10–14]. In children and adolescents, the most recent literature has reported symptoms arising from disorders such as pseudohypacusis and functional upper airway obstruction [15–17]. Paradoxical vocal cord motion, or psychogenic stridor, refers to the inappropriate adduction of the vocal cords during the respiratory cycle, and remains a common and frequently misdiagnosed functional disorder in the pediatric population. Over 50% of patients with paradoxical vocal cord motion are diagnosed with conversion disorder [12]. The differential diagnosis in the pediatric population is challenging, given the high base rates of pediatric mental health disorders including conversion disorder, adjustment disorder and autism spectrum disorder. This has the potential to be mistakenly diagnosed as primary otolaryngologic disease, as observed in this case series.

Conversion disorder (CD), or functional neurological symptom disorder, is characterized by disturbances in body function that are inconsistent with known anatomy or pathophysiology [18]. The Diagnostic and Statistical Manual of Mental Disorders V defines the conversion disorder as the presence of “one or more symptoms of altered voluntary motor or sensory function” in the absence of any identifiable neurological or medical cause. While the symptoms are psychogenic in origin, conversion disorder distinguishes itself from malingering and factitious disorder as the CD patient is not intentionally experiencing these symptoms [18, 19]. The patient population affected can be characterized as having perfectionist tendencies with high expectations regarding achievements and high levels of anxiety associated with illness [20–25]. There is no clear etiology of conversion disorder. However, in general, theories focus on the management of affect and stress [5, 18, 20–22, 24, 25].

There are no bedside tests or investigations to establish the diagnosis of conversion disorder. The diagnosis is made after organic disease has been ruled out [5, 18]. As such, diagnosing conversion disorder can put the clinician in the difficult position of having to communicate the presence of a non-organic illness as the source of the patient’s severe disability, while validating the authenticity of their symptoms [20]. However, this communication combined with appropriate intervention assists in validating symptoms and provides a mechanism for their resolution.

Although it is rare for the practicing surgical specialist to encounter this disorder, its atypical sensory and motor manifestations make it a potential diagnosis in any specialist’s practice. For instance, these patients may find themselves under the care of an Otolaryngologist-Head and Neck Surgeon to rule out organic hearing loss or peripheral vertigo as the etiology of their symptoms [26]. Objective tests of hearing and vestibular dysfunction, including audiometry, electronystagmography and rotational chair testing are essential to rule out organic pathology. Ancillary studies to detect non-organic pathology in children, including the Stenger Test, can be considered to identify pseudohypoacusis. The authors encourage consideration of psychological stressors as factors to be considered, which may be associated with conversion, or other, disorders encountered in severe disease and in the postoperative period as demonstrated in the presented cases. Early diagnosis and therapy can significantly improve health outcomes in these patients [19], and the prognosis is considered to be excellent (with roughly 95% of affected individuals experiencing spontaneous resolution of their symptoms within one month of diagnosis) [27].

## A diagnostic challenge

### Understanding the patient

With the support of online resources and media publicity, patients now present with a plethora of computer-generated differential diagnoses and planned diagnostic investigations independent of their physician’s input [28]. However, the new age of knowledgeable and autonomous patients poses both benefits and challenges to clinicians.

Patients may find it challenging to accept a psychiatric diagnosis, which is based heavily on clinical judgment, when they have initially become invested in the concept of an organic disease as the etiology of their symptoms. The agnostic approach, where possible diagnoses and explanations are equally valid, is used by clinicians to manage relaying a lack of diagnosis to a family [29]. This approach may avoid questions about the authenticity of symptoms that can contribute to a hostile physician-patient relationship [20, 29]. Clinicians should be aware of the very sensitive and frank discussion that needs to take place with patients regarding the nature of their illness. Establishing a forum for discussion will also assist in providing guidance around patient-specific therapy for this disorder [20]. This discussion, validating both physical and mental symptoms, optimally could include an open invitation for the value of mental health professionals as part of the health care team.

### Superfluous diagnostic testing

Diagnosis of mental illness continues to rely heavily on clinical judgment and judicious use of diagnostic testing. This means that clinicians must balance patient perspectives and values with clinical practice guidelines and professional expertise. This can be challenging in the context of unexplained symptoms and patients’ conceptualizations about the symptoms, but optimally can be an opportunity to assist the patient in accessing evidence-based resources and care.

The patient-physician relationship is founded heavily in trust from both parties. This may invoke some restraint on the part of the clinician in opposing patient requests for non-beneficial investigations and procedures [28]. However, clinicians must be wary of the fact that both patients as well as physicians can introduce biases into clinical decision-making that can complicate care and increase health care costs [30]. Brett and McCullogh [28, 31] described an approach to clinical practice in the context of differing interests to facilitate patient-physician relationships. The authors recommend that a patient’s preference for a diagnostic or therapeutic intervention dictate medical decision-making only when there is a modicum of potential clinical benefit. It is only if they meet this criterion that physicians proceed with patient-selected interventions [28, 31].

In addition to their patients, clinicians also have a fiscal responsibility to the health care system on a societal level to ensure its sustainability and judicious use of its resources [28, 32]. Although cost should not prevent patients from receiving optimal medical care, the fact remains that non-beneficial interventions have implications for individual patients and society as a whole and should be considered essential to professional integrity [28, 33–35]. Eliminating waste in diagnostic interventions, including duplicate and non-beneficial testing, can reduce a significant cost burden on the health care system [33]. At a certain threshold, challenging a patient’s request when supported by clinical evidence should not be misconstrued as a denial of patient’s perspective, but rather a professional responsibility to ensure cost-effective medical care [28]. This is best managed through transparent and collaborative dialogue, focused on the value of further investigations in improving the understanding of the evolving clinical picture and/or changing the clinical management.

### Approach to the atypical otolaryngology-head and neck surgery patient

The appropriate management of pediatric patients with atypical symptoms can be a challenging task for Otolaryngologists. This report should stand as a resource for clinicians who encounter difficult cases in this context (Table 1). Open dialogue should be maintained with the patient and their family throughout the patient interaction. In challenging cases, such as those presented in this article, it is important for clinicians to broaden their differentials to include non-organic etiologies of otolaryngology disorders. Evaluation of these patients should be initiated with a thorough history and should note any potential risk factors for mental health and psychosocial factors which may impact the resiliency of the patient to cope with medical interventions, including surgery. Physical examination should include a complete oto-neurologic examination and test of vestibular function. Rehabilitation should be prescribed based on results of screening and diagnostic testing. A consultation with Neurology and Mental Health services should be contemplated early, and where appropriate, an integrated team approach considered. The final care plan for these patients should commence with an understanding of the patient and family’s goals, validation of the patient’s symptomology and appropriate plan of care.

**Table 1:** Approach to the atypical ENT patient

Establish broad differential diagnoses^a^	
Organic diseases	
Non-organic diseases	Pseudohypacusis
	Functional upper airway obstruction
	Conversion disorder
	Adjustment disorder
	Autism spectrum disorders
Patient evaluation	Thorough history and physical examination including flexible nasolaryngoscopy
	Audiometry and vestibular testing should be performed to rule out organic pathology as indicated by the presenting complaint
	Consultation with relevant specialists including neurology and psychiatry
	Consider neuroimaging to rule out structural pathology
Treatment overview	Assessment of goals with staff, patient and family
	Confirm belief in presenting symptoms
	Avoid accusation
	Ensure patient and family is connected with appropriate community resources including physical or psychological rehabilitation
	Arrange follow-up visits

^a^ This should be a resource utilized by clinicians in appropriate cases where an organic pathology has been ruled out

## Conclusion

Delayed identification of mental illness can result in significant medical and psychological consequences for patients, increase the burden of care, and can impact their faith in the health care system. Furthermore, it creates a significant economic burden for the health-care system as a whole. In this case series, the authors present 3 patients in whom presentation and management of otolaryngology-related concerns were confounded by underlying conversion disorder.

While most patients referred to an Otolaryngology-Head and Neck Surgeon will have organic explanations for their symptoms, it is important for the clinician to keep psychogenic causes and contributors on the differential, especially in patients with atypical clinical presentations. The diagnostic approach to these patients requires a comprehensive assessment of the contributing factors, including the features of converson disorder associated with otolaryngology diseases, and impact the relationship between patients and clinicians. This article is intended to stimulate discussion between patients and clinicians regarding safe and efficient diagnosis of challenging clinical cases.

## Abbreviations

CD: Conversion disorder
CT: Computed tomography
MRI: Magnetic resonance imaging
